# Hypoxia-induced Wnt/β-catenin signaling activation in subchondral bone osteoblasts leads to an osteoarthritis-like phenotype of chondrocytes in articular cartilage

**DOI:** 10.3389/fmolb.2023.1057154

**Published:** 2023-04-21

**Authors:** Fang Li, Qizhao Tan, Feng Li, Ke Zhang, Zhongjun Liu, Yun Tian, Tengjiao Zhu

**Affiliations:** ^1^ Department of Hematology, Peking University Third Hospital, Beijing, China; ^2^ Department of Orthopaedics, Peking University Third Hospital, Beijing, China; ^3^ Department of Orthopaedics, Zibo Central Hospital, Zibo, Shandong, China; ^4^ Engineering Research Center of Bone and Joint Precision Medicine, Ministry of Education, Beijing, China; ^5^ Department of Orthopaedics, Peking University International Hospital, Beijing, China

**Keywords:** osteoarthritis, subchondral bone, articular cartilage, hypoxia, Wnt pathway, crosstalk

## Abstract

**Background:** Osteoarthritis (OA) is a whole-joint disease and characterized by alterations in the articular cartilage, subchondral bone, ligaments, and synovial membrane. The crosstalk between cartilage and subchondral bone plays a crucial role in the pathogenesis and progression of OA. Hypoxia has been reported to play an important role in cartilage degradation and subchondral bone remodeling in OA. In this study, we aimed to identify the involvement of hypoxia in modifying the osteoblast phenotypes and determine whether these alterations could influence the metabolism of chondrocytes.

**Methods:** First, the levels of Hif-1α in subchondral bone of different compartments in patients with OA were assessed using immunohistochemistry (IHC). In *in vitro*, human primary osteoblasts were cultured under hypoxic and normoxic conditions, and the hypoxic or normoxic conditioned media (HCM and NCM) were used to culture human primary chondrocytes. Then, phenotypic changes in osteoblasts were assessed using reverse transcription-polymerase chain reaction (RT-PCR), Western blotting, and enzyme-linked immunosorbent assay (ELISA). Furthermore, the expression of type II collagen (COL2A1), aggrecan (ACAN), SRY-related high-mobility group-box gene 9 (SOX9), matrix metalloproteinase 13 (MMP13), and matrix metalloproteinase 3 (MMP3) in chondrocytes was measured using RT-PCR. Finally, the serum levels of Wnt-related proteins were determined using ELISA.

**Results:** Hif-1α was significantly increased in severely sclerotic subchondral bone compared to less damaged subchondral bone. β-Catenin and SOST were identified as upregulated and downregulated in hypoxic osteoblasts, respectively. The hypoxia-induced results were confirmed by ELISA. Stimulating human primary chondrocytes with HCM significantly induced MMP13 and MMP3 and inhibited COL2A1, ACAN, and SOX9 mRNA expression. The serum levels of DKK-1 were significantly increased in human OA.

**Conclusion:** Together, these findings revealed that hypoxia in subchondral bone is a key factor in the crosstalk between chondrocytes and osteoblasts and facilitates the shift of chondrocytes toward an OA-like phenotype probably by activating the Wnt/β-catenin signaling pathway in osteoblasts.

## Introduction

Osteoarthritis (OA) is the most common joint disease and a major cause of pain and disability in the elderly. The degeneration of articular cartilage is classically considered to be the central pathological feature of OA (Felson and Neogi, 2004); however, this widely accepted notion has been challenged by recent studies that emphasize the pathophysiological role of the subchondral bone.

The close anatomic association between cartilage and subchondral bone makes molecular crosstalk between them possible. Cumulative data have recently shown that calcified cartilage contains unmineralized regions (Pan et al., 2009), allowing the transfer of soluble products between subchondral bone and articular cartilage. However, aberrant molecular communications between overlying cartilage and the subchondral bone are a critical aspect in the OA process (Yuan et al., 2014).

Alterations in subchondral bone and articular cartilage are regulated by the bone cells and chondrocytes. In response to altered biochemical and mechanical environments in the course of OA, these cells exhibit different patterns of gene expression (Findlay and Atkins, 2014). Intercellular communications between cells in subchondral bone and chondrocytes are able to maintain the joint homeostasis (Zhang et al., 2020), and disruption of cellular communications might lead to joint disorders such as OA. An appreciation of altered cellular phenotypes and their interactions in OA might be helpful in identifying new strategies for OA-targeted therapy.

In OA, decreased oxygen tension in both the synovial fluid and synovium was demonstrated (Liu et al., 2019). Similar to the aforementioned results, studies also found that subchondral bone from individuals with OA displays abnormal arterial and venous perfusion kinetics compared to that from normal controls in both animal and human studies (Lee et al., 2009; Dyke et al., 2015; Aaron et al., 2018), and the vascular occlusion and venous stasis may result in local hypoxia (Luyendyk et al., 2005; Chang et al., 2014). Recently, accumulating evidence reveals that hypoxia exists in subchondral bone in the OA process (Chang et al., 2014; Li et al., 2020; Ching et al., 2021). The disruption of oxygen supplement could trigger a cascade of responses at both the molecular and cellular levels. These findings suggest that hypoxia of subchondral bone plays a vital role in the process of OA. However, few studies have reported the role of hypoxia in regulating the molecular alterations in subchondral bone. Additionally, whether hypoxia can influence the crosstalk between subchondral bone and articular cartilage remains unclear.

In this study, we hypothesized that the distinctive pro-catabolic chondrocyte phenotype observed in OA may be induced by biochemically soluble factors produced by subchondral bone cells responding to hypoxia. Using a conditioned medium from hypoxic osteoblasts and conditioned medium from normoxic osteoblasts, we demonstrated that soluble mediators released by hypoxic osteoblasts were able to induce a chondrocyte OA-like phenotype. These results may provide new insights into the pathogenesis of OA and the discovery of promising drug targets for OA treatment.

## Materials and methods

### Clinical sample collection

The tibial plateau was harvested arthroscopically from 17 patients with OA who underwent total knee replacement surgery (TKA) in Peking University International Hospital. These patients were enrolled after they met the following criteria: above 65 years old with obvious varus deformity of the knee joint without osteoporosis or undergoing anti-osteoporosis treatment. The subchondral bone in the medial tibial plateaus had marked sclerosis, and the subchondral bone in the lateral tibial plateaus was less damaged. The bone sclerosis is observed as described previously (Janelle-Montcalm et al., 2007). The subchondral bone in the medial tibial plateaus was defined as the AS group, and the subchondral bone in the lateral plateaus was defined as the NS group. This study was approved by the Ethics Committees of Peking University International Hospital, and all patients signed the informed consent.

### Immunohistochemistry assessment of clinical samples

IHC was performed as described previously (Cui et al., 2018). Briefly, the sections were then deparaffinized in graded alcohol solutions, and antigen retrieval was performed using gastric enzymes at 37°C for 20 min. The tissue sections were incubated at 4°C overnight with primary antibodies against hypoxia-inducible factor 1 (Hif-1) (dilution 1:100; Cat No.: ab2185, Abcam, Cambridge, MA, United States) and β-catenin (dilution 1:50; Cat No.: A5038, Bimake, Houston, TX, United States), followed by incubation with secondary antibodies against biotin (Cat No.: PV-6001, ZSGB-BIO, BJ, China) for 30 min at 25°C. Bound antibodies were visualized using a chromogen diaminobenzidine (DAB) kit (ZSGB-BIO, Beijing, China). Images were captured using a digital slide scanner (Hamamatsu, Japan) and semi-quantitatively analyzed using Image-Pro Plus (v.6.0, Media Cybernetics, Rockville, MD, United States). The staining intensity was estimated using the mean integrated optical density (IOD) of three randomly selected high-power fields per sample.

### Isolation and culture of human OA primary osteoblasts and chondrocytes

Primary osteoblasts were isolated from the early-stage subchondral bone of the lateral tibial plateau in human OA by successive enzymatic digestion, as described previously (Lajeunesse et al., 2004). Briefly, early-stage subchondral bone samples were cut into small pieces (∼1 mm^2^) before being sequentially digested in the presence of 1 mg/mL collagenase type I (Cat No.: jym-1, Sigma-Aldrich, St. Louis, MO, United States) in Dulbecco’s modified Eagle’s medium (DMEM) (Gibco, Grand Island, NY, United States) without serum at 37°C for two periods of 30 and 240 min. Digested subchondral bone pieces were then washed with the same medium and cultured in DMEM containing 10% fetal bovine serum (FBS) (Gibco) and 1% penicillin–streptomycin. This medium was replaced every 2 days for 28–35 days until 90% confluence was reached. Only first-passage cultured OA osteoblasts were used in this study.

Primary chondrocytes were isolated from the relative intact cartilage of the lateral tibial plateau in human OA by sequential enzymatic digestion, as described previously (Reboul et al., 2001) and cultured in DMEM supplemented with 10% FBS and 1% penicillin–streptomycin. The medium was replaced every 2 days for 14 days until 90% confluence was reached. Only first-passage OA chondrocytes were used in this study.

### Hypoxic culture of OA primary osteoblasts

After human primary osteoblasts were cultured in 10-cm plates (50,000 cells/cm^2^) in DMEM for 28–35 days, cells from each patient (*n* = 6) were divided into four 6-cm plates by trypsin digestion. Two 6-cm plates were cultured under normoxia (21% oxygen) in a standard humidified incubator at 37°C with 5% CO_2_/95% air for 12 or 24 h. For the hypoxia experiments, two plates were moved into humidified incubators at 37°C with 5% CO_2_, and the oxygen tension was reduced to 1% separately for 12 and 24 h. The culture supernatant was retained for chondrocyte stimulation and ELISA, while the osteoblasts were harvested for RNA and protein extraction. Primary cells from different patients were not mixed due to heterogeneity.

### Stimulation of human OA primary chondrocytes with conditioned media

After human primary chondrocytes were cultured in 10-cm plates (50,000 cells/cm^2^) in DMEM for 14 days, cells from each patient (*n* = 6) were divided into eight 6-cm plates by trypsin digestion. Two plates were cultured with 0.5 mL DMEM and 0.5 mL conditioned medium from hypoxia OA subchondral bone osteoblasts (HCM) for 12 h, and two plates were cultured with 0.5 mL DMEM and 0.5 mL HCM for 24 h. The remaining four plates were cultured with 0.5 mL DMEM and 0.5 mL conditioned medium from OA normoxia subchondral bone osteoblasts (NCM) for either 12 or 24 h. The chondrocytes stimulated with HCM and NCM for 12 and 24 h were retained and harvested for RNA extraction.

### Reverse transcription-polymerase chain reaction (RT-PCR)

The total RNA was extracted from stimulated human chondrocytes and osteoblasts under hypoxia or normoxia and reverse-transcribed into cDNA using a FastKing First Strand cDNA Synthesis Kit (TIANGEN, Cat No.: KR118, BJ, China) according to the manufacturer’s instructions. Quantitative real-time PCR was performed in triplicate, and the relative expression of target genes was calculated by using the Livak method and normalized to β-actin. The gene-specific primers used are shown in Table 1.

**TABLE 1:** Primer sequences used for quantitative real-time PCR.

Gene	Sequence (5′-3′)	
COL2A1	Forward	TGG​ACG​ATC​AGG​CGA​AAC​C
	Reverse	GCT​GCG​GAT​GCT​CTC​AAT​CT
SOX9	Forward	AGC​GAA​CGC​ACA​TCA​AGA​C
	Reverse	CTG​TAG​GCG​ATC​TGT​TGG​GG
ACAN	Forward	CCC​CTG​CTA​TTT​CAT​CGA​CCC
	Reverse	GAC​ACA​CGG​CTC​CAC​TTG​AT
MMP13	Forward	CCA​GAC​TTC​ACG​ATG​GCA​TTG
	Reverse	GGC​ATC​TCC​TCC​ATA​ATT​TGG​C
MMP3	Forward	CTG​GAC​TCC​GAC​ACT​CTG​GA
	Reverse	CAG​GAA​AGG​TTC​TGA​AGT​GAC​C
HIF-1α	Forward	ATC​CAT​GTG​ACC​ATG​AGG​AAA​TG
	Reverse	TCG​GCT​AGT​TAG​GGT​ACA​CTT​C
SOST	Forward	ACA​CAG​CCT​TCC​GTG​TAG​TG
	Reverse	GGT​TCA​TGG​TCT​TGT​TGT​TCT​CC
CTNNB1	Forward	AAA​GCG​GCT​GTT​AGT​CAC​TGG
	Reverse	CGA​GTC​ATT​GCA​TAC​TGT​CCA​T

### Western blotting

Four 6-cm plates of 12 h hypoxic osteoblasts and four 6-cm plates of 12 h normoxic osteoblasts were washed twice with cold phosphate-buffered saline (PBS), lysed with RIPA, and then incubated on ice and vortexed for 30 s every 10 min for a total of three times. The supernatant was collected after centrifugation (12,000 rpm for 10 min at 4°C), and the protein concentration of the solution was determined using a BCA kit (Beijing Solarbio Science and Technology, Beijing, China). The total protein (40 μg) was subjected to 10% sodium dodecyl sulfate polyacrylamide gel electrophoresis (SDS-PAGE) and blotted onto polyvinylidene fluoride (PVDF) membranes (Millipore, Bedford, MA, United States). The membranes were then incubated with primary antibodies against β-catenin and β-actin (1:1000 dilution), and the results were detected using an Odyssey detection system (LI-COR Biosciences, Lincoln, NE, United States).

### ELISA

HCM (*n* = 6) and NCM (*n* = 6) from 12 h human primary osteoblasts were collected and stored at −80°C. β-Catenin and sclerostin (SOST) concentrations were quantified using commercially available test kits according to the manufacturer’s instructions (Cat Nos: MM-2451H1 and MM-50942H1, respectively, Meimian, Yancheng, China). Blood samples were obtained from patients with OA (*n* = 28) before TKA and non-OA controls (*n* = 22) before arthroscopic surgery. Serum was separated and stored at −80°C before β-catenin, SOST, and Dickkopf-related protein (DKK-1) concentrations were quantified using commercially available test kits according to the manufacturer’s instructions (Cat Nos: MM-2451H1, MM-50942H1, and, MM-50352H1, respectively, Meimian).

### Statistical analysis

Data are presented as the mean ± standard deviation (SD). The unpaired Student’s *t*-tests between two groups and one-way ANOVA among three groups were used to compare the data. Significance levels were set at *p < 0.05, **p < 0.01, and ***p < 0.001. All data were analyzed using SPSS software (v.19.0).

## Results

### Hif-1α are differentially expressed in AS and NS

**FIGURE 1:**
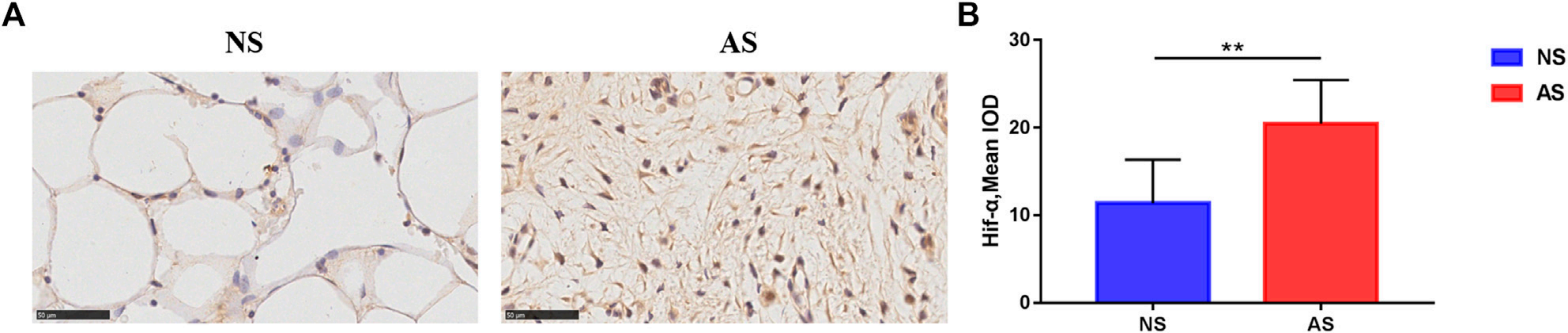
Immunohistochemistry results of tibial plateau samples. **(A)** Representative images of Hif-1α expression in the NS and AS groups. Scale bars represent 500 μm. IOD, integrated optical density. **(B)** Semi-quantitative analysis of Hif-1α expression in the NS and AS groups. NS, less damaged subchondral bone; AS, severely damaged subchondral bone.

First, we explored the expression of Hif-1α, a known biomarker of hypoxia, in the subchondral bone of different compartments by IHC analysis. As shown in Figure 1A, we observed more Hif-1α-positive stained cells in the AS group, with quantitative analysis demonstrating a significant increase in Hif-1α expression in the AS group (Figure 1B), suggesting that tissue hypoxia occurs in AS.

### Hypoxia regulates Wnt-related genes and proteins in osteoblasts

**FIGURE 2:**
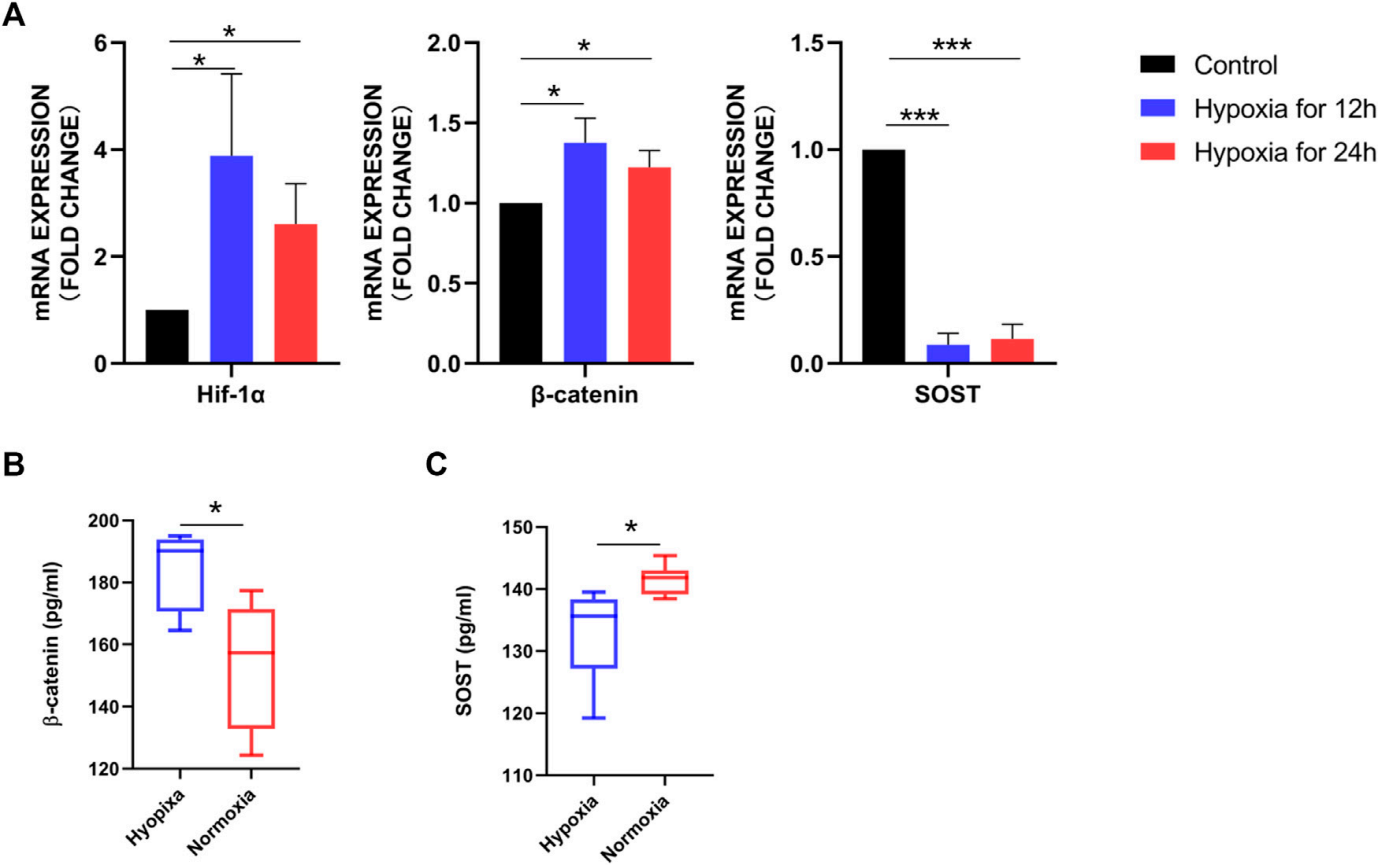
Differentially expressed soluble mediators identified in osteoblasts under hypoxia. **(A)** RT-PCR analysis of Hif-1α, β-catenin, and SOST expression. Hif-1α and β-catenin expression was significantly higher in osteoblasts cultured under hypoxia for 12 and 24 h than under normoxia for 12 and 24 h. **(B)** Western blot analysis demonstrating increased β-catenin expression in osteoblasts after 12 h hypoxia, with β-actin as the loading control. **(C)** β-Catenin and SOST concentrations in the conditioned medium of osteoblasts cultured under hypoxia and normoxia for 12 h detected using ELISA.

To determine whether hypoxia would affect the phenotype of osteoblasts, we assessed the expression of selective gene targets. Compared to osteoblasts cultured under normoxia for 12 and 24 h, Hif-1α (*p* < 0.05) and β-catenin (*p* < 0.001) mRNA levels were significantly increased in osteoblasts cultured under hypoxia for 12 and 24 h, whereas SOST (*p* < 0.05) levels were significantly decreased (Figure 2A). The remarkedly increased β-catenin levels in osteoblasts under hypoxia compared to those under normoxia were confirmed by Western blot analysis (Figure 2B).

In addition, we measured β-catenin and SOST concentrations in the supernatant of osteoblasts cultured under hypoxia and normoxia for 12 h using ELISA. Consistent with the results of PCR and Western blotting, the median value of β-catenin was higher in the HCM (184.34 pg/mL) than NCM (153.56 pg/mL; *p* = 0.0103), whereas SOST levels were significantly lower in HCM than NCM (132.96 *vs*. 141.52 pg/mL, *p* = 0.0238) (Figure 2C).

### HCM from osteoblasts induces a chondrocyte OA-like phenotype

**FIGURE 3:**
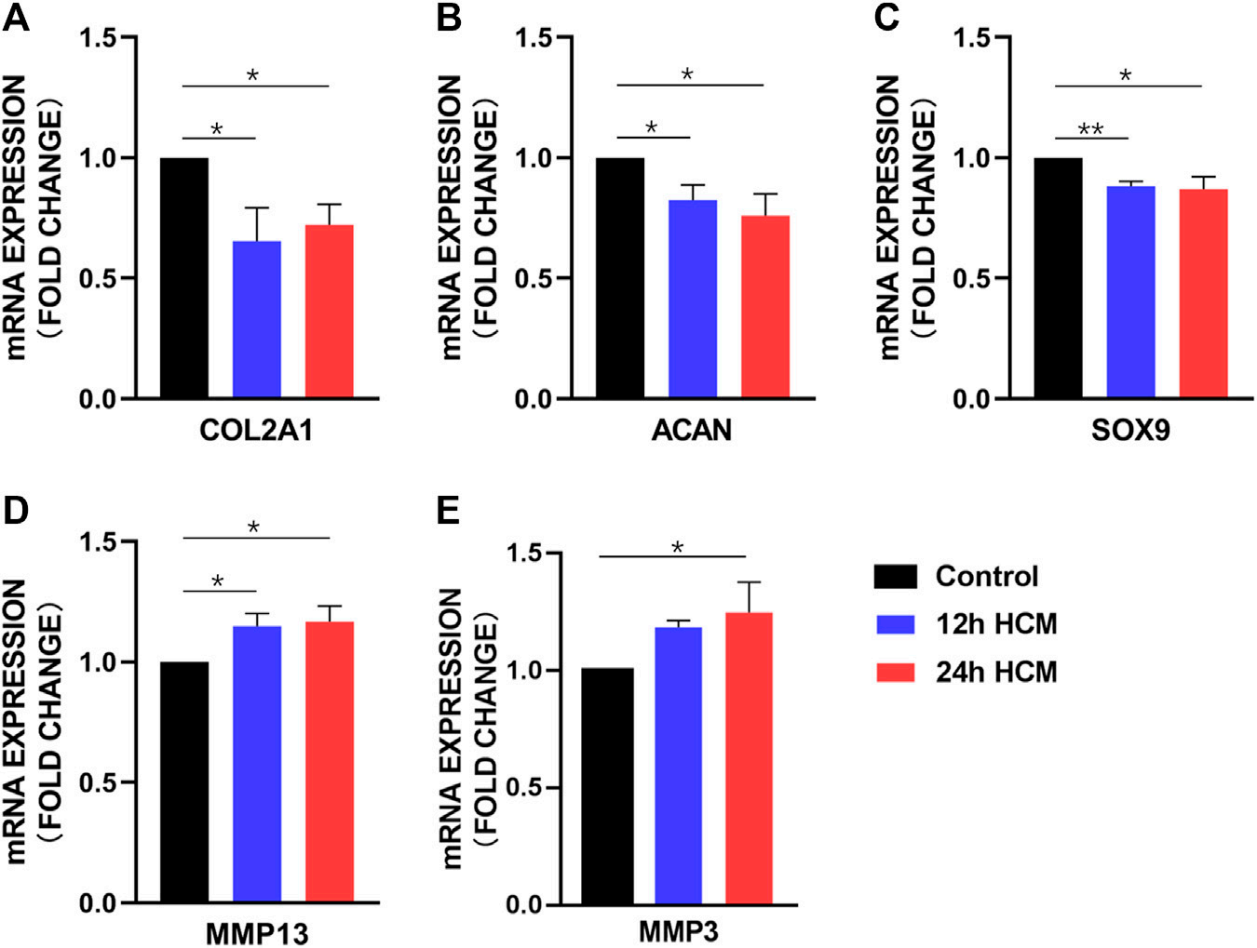
RT-PCR analysis of the osteoarthritis biomarker mRNA levels in human primary chondrocytes stimulated with a conditioned medium from hypoxia (HCM) for 12 h. **(A)** COL2A1, **(B)** ACAN, and **(C)** SOX9 expression was significantly decreased in human primary chondrocytes stimulated with 12 and 24 h HCM compared to the 12 and 24 h conditioned medium from normoxia (NCM). **(D)** MMP13 expression was significantly increased in human primary chondrocytes stimulated with 12 and 24 h HCM compared to 12 and 24 h NCM, respectively. **(E)** MMP3 expression was significantly increased in human primary chondrocytes stimulated with 12 h HCM compared to those stimulated with 12 h NCM but not 24 h HCM compared to 24 h NCM.

To determine whether HCM caused phenotype changes in primary chondrocytes, we next analyzed changes in the expression of the following typical biomarkers of OA in chondrocytes: type II collagen (COL2A1), aggrecan (ACAN), SOX9, MMP13, and MMP3. Primary chondrocytes stimulated with HCM for 12 h displayed significantly decreased COL2A1, ACAN, and SOX9 expression (Figures 3A–C) and significantly increased MMP13 and MMP3 expression (Figures 3D, E) compared to those stimulated with 12 h NCM. Similarly, primary chondrocytes stimulated with HCM for 24 h exhibited significantly decreased COL2A1, SOX9, and ACAN expression (Figures 3A–C) and significantly increased MMP13 and MMP3 expression (Figures 3D, E) compared to those stimulated with 24 h NCM.

**FIGURE 4:**
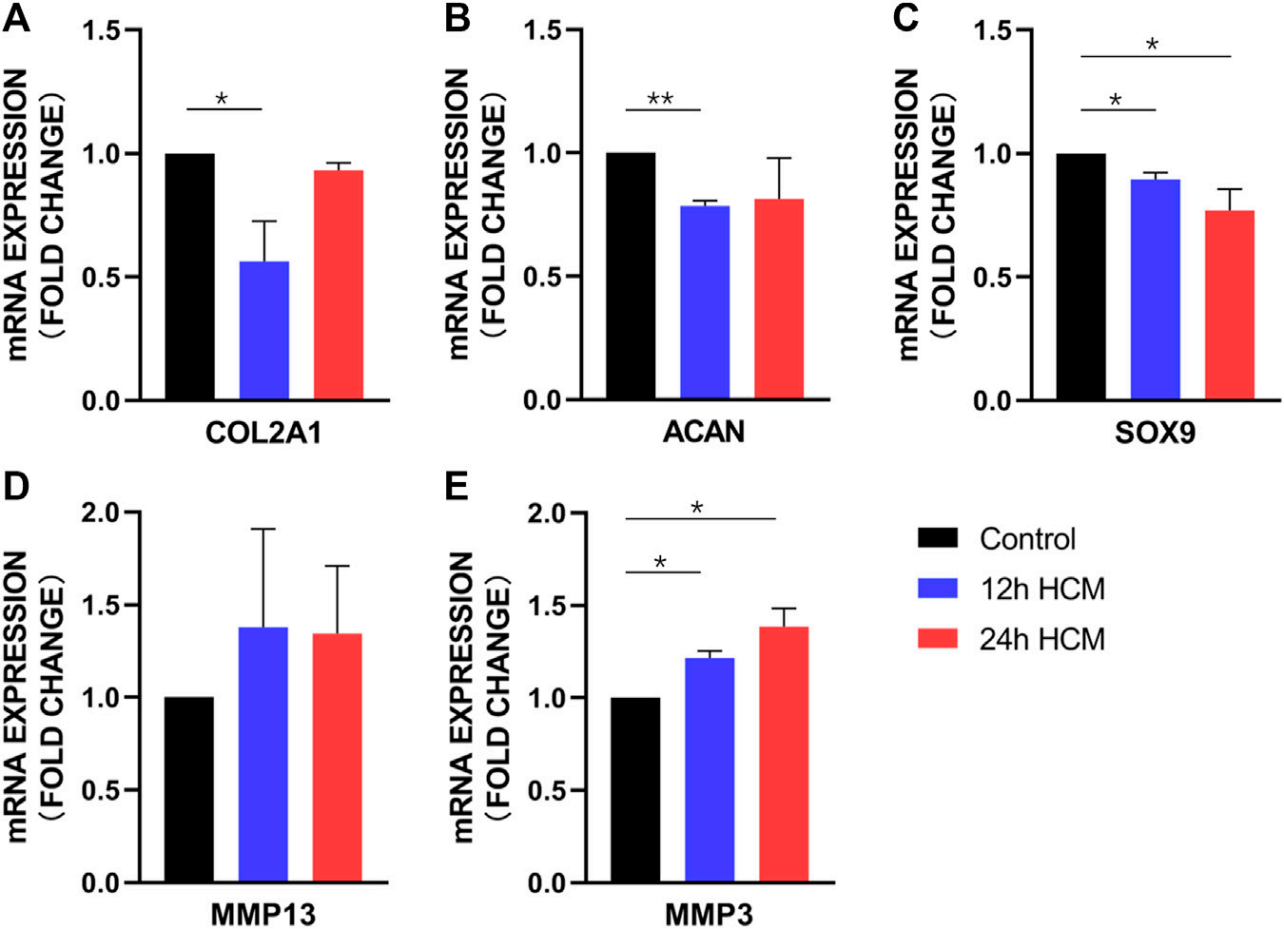
RT-PCR analysis of osteoarthritis biomarker mRNA levels in human primary chondrocytes stimulated with a conditioned medium from hypoxia for 24 h. **(A)** COL2A1, **(B)** ACAN, and **(C)** SOX9 expression was significantly decreased in human primary chondrocytes stimulated with 12 h HCM compared to those stimulated with 12 h conditioned medium from normoxia. **(A)** COL2A1, **(B)** ACAN, and **(C)** SOX9 expression was decreased in human primary chondrocytes stimulated with 24 h HCM compared to those stimulated with 24 h NCM, with a significant difference in SOX9 expression but not in COL2A1 and ACAN expression. **(D)** MMP1 and **(E)** MMP3 expression was increased in human primary chondrocytes stimulated with 12 h and 24 h HCM compared to those stimulated with 12 and 24 h NCM, with a significant difference in MMP3 expression but not MMP13 expression.

Primary chondrocytes stimulated with 12 h HCM for 24 h displayed significantly lower COL2A1, SOX9, and ACAN expression than those stimulated with 12 h NCM (Figures 4A–C), whereas MMP3 expression significantly increased (Figure 4E) and MMP13 expression did not change significantly (Figure 4D). When primary chondrocytes were stimulated with 24 h HCM for 24 h, they exhibited significantly decreased SOX9 expression and significantly increased MMP3 expression compared to stimulation with 24 h NCM (Figures 4C, E); however, no significant changes in COL2A1, ACAN, or MMP13 expression were observed (Figures 4A, B, D).

#### β-Catenin is differentially expressed in the AS and NS groups

**FIGURE 5:**
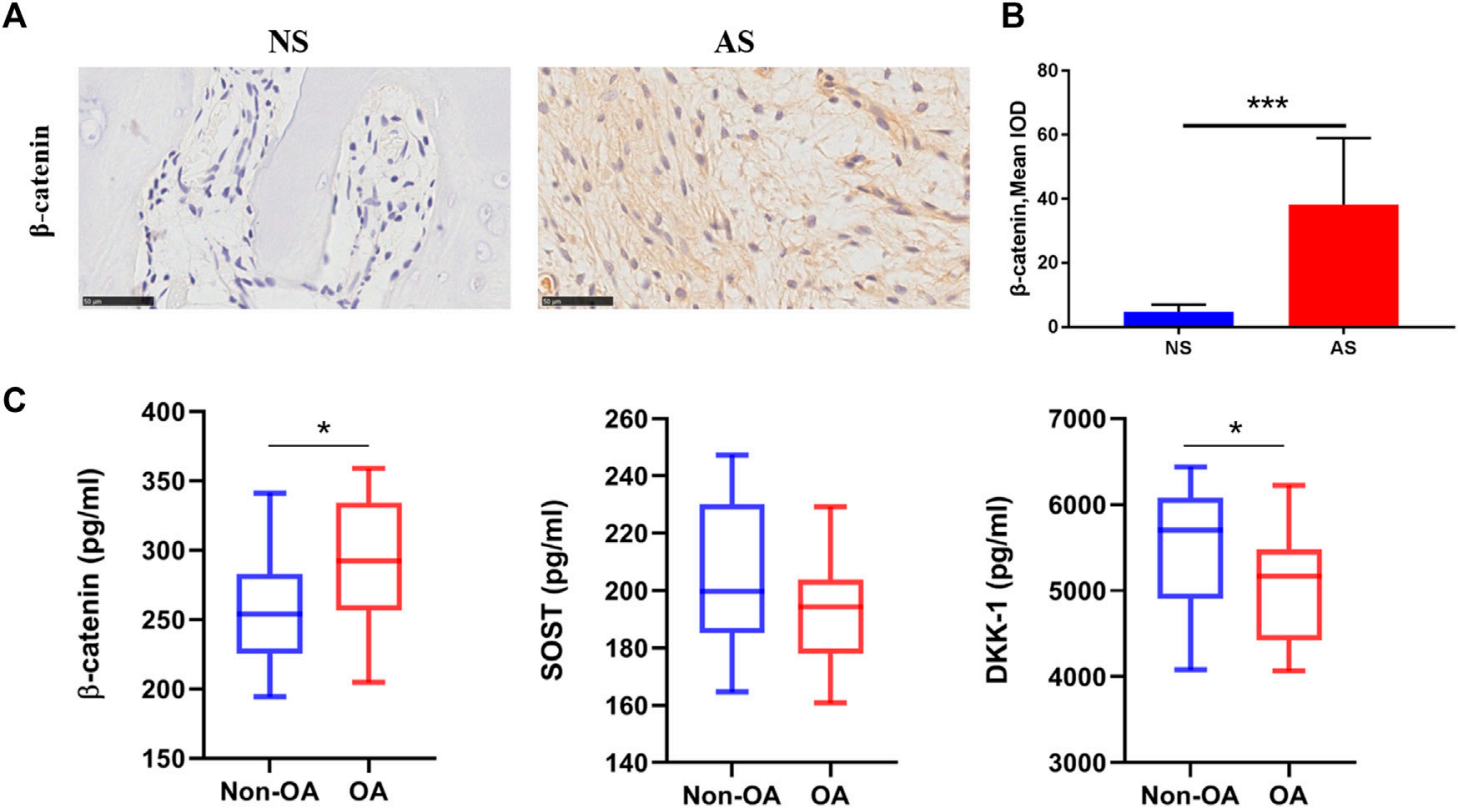
Wnt-related protein expression in clinical samples. **(A)** Representative images of β-catenin expression in tibial plateau samples from the NS and AS groups. Scale bars represent 500 μm. **(B)** Semi-quantitative analysis of the β-catenin expression in the NS and AS groups. **(C)** β-Catenin, SOST, and DKK-1 serum concentrations in patients with and without osteoarthritis. NS, less damaged subchondral bone; AS, severely damaged subchondral bone; IOD, integrated optical density.

To further confirm the activity of Wnt signaling in subchondral bone in the OA process, we performed IHC analysis. More positively stained cells were observed in the AS group (Figure 5A) and the semi-quantitative result showed that the expreesion level of β-catenin in the AS group was higher than the NS group (Figure 5B). The finding was in line with cell hypoxia experiments.

#### β-Catenin, SOST, and DKK-1 are differentially expressed in the serum of patients with and without OA

Lastly, we further measured the serum levels of β-catenin, SOST, and DKK-1 using ELISA to detect any correlation between Wnt/β-catenin signaling and the clinical diagnosis of OA in patients. Patients with OA displayed higher serum β-catenin levels (288.10 *vs*. 263.72 pg/mL, *p* = 0.0631) with marginal statistical significant difference and lower serum SOST levels (193.24 *vs*. 203.41 pg/mL, *p* = 0.1138) with no significant difference compared to those in the non-OA control group. Interestingly, the OA group also had significant lower serum levels of DKK-1 (5042.15 pg/mL), a typical Wnt/β-catenin signaling antagonist, than the control group (5494.42 pg/mL; *p* = 0.0258; Figure 5C).

## Discussion

In the study, first, hypoxia in the severely damaged subchondral bone was determined. Thereafter, human primary osteoblasts derived from subchondral bone were subjected to hypoxia or normoxia, and then, their conditioned media were used to stimulate human primary chondrocytes *in vitro*. We found that HCM can lead to a chondrocyte OA phenotype by upregulating the expression of proteases (MMP3 and MMP13) and downregulating COL2A1, SOX9, and ACAN expression. These findings, for the first time, indicate that the phenotype of chondrocytes can be regulated by subchondral osteoblasts in response to hypoxia and the subchondral hypoxia may play detrimental roles in cartilage hemostasis.

Hif-1α, a biomarker of hypoxia, was found to be increased in severely damaged subchondral bone compared to less damaged subchondral bone in patients with OA. The finding suggests that the levels of hypoxia might be different at different stages of OA, and the severely damaged subchondral bone in the late-stage OA shows the existence of more severe hypoxia. Moreover, the results indicate that the subchondral bone hypoxia has already been implicated in the pathogenesis of OA and the levels of hypoxia can distinguish the severity of subchondral bone destruction. A previous study has shown that hypoxia and Hif-1α expression was reduced in destabilization-induced mice OA cartilage compared with control mice (Bouaziz et al., 2016). The contradiction may be explained by the fact that the cartilage is physiologically in a hypoxic state, whereas the subchondral bone has more blood supplement, and the oxygen tension in these two different tissues may be different in the OA process.

RT-PCR, Western blotting, and ELISA were performed on human primary osteoblasts to identify alterations in osteoblast phenotypes in response to hypoxia. Based on PCR analysis, both Hif-1α and β-catenin were significantly upregulated in osteoblasts under hypoxia, whereas SOST, a typical antagonist of Wnt signaling, was significantly downregulated. Western blotting further confirmed that β-catenin was significantly upregulated in osteoblasts after hypoxia. β-Catenin and SOST levels in the supernatant were consistent with those in the PCR analysis. β-Catenin is a key molecule in the Wnt/β-catenin signaling pathway that is distributed in the cell membrane, cytoplasm, and nucleus (Bodine, 2008) and is closely related to Wnt signaling activation. SOST, specifically expressed in the bone tissues of adults (van Bezooijen et al., 2004), is considered to be a typical Wnt signaling antagonist (Bouaziz et al., 2015; Li et al., 2019). Therefore, these results suggest that hypoxia leads to the activation of Wnt/β-catenin signaling in osteoblasts.

Additionally, it is acknowledged that Hif-1α can enhance Wnt/β-catenin signaling (Stegen et al., 2018); the increased transcription level of Hif-1α strengthens the notion that Wnt/β-catenin signaling activation takes place in subchondral bone osteoblasts in response to hypoxia. To further confirm Wnt/β-catenin signaling activation during the pathophysiological development of OA, we also investigated β-catenin expression in subchondral bone of the human OA tibial plateau. As expected, the markedly increased β-catenin expression in severely damaged subchondral bone was confirmed using IHC. Given the vital role of β-catenin to drive Wnt signaling, we provide further evidence suggesting that subchondral bone tissue in patients with OA underwent the activation of the Wnt pathway.

One of the hallmark findings of this study is that the HCM from osteoblasts under the hypoxia environment can cause an OA phenotype of chondrocyte, which indicates hypoxia results in a detrimental crosstalk between chondrocytes and osteoblasts. A previous study demonstrated that osteoblasts from the sclerotic areas of the OA subchondral bone induced chondrocyte catabolic activity (Sanchez et al., 2005). Our results have shown that under hypoxic environment, osteoblasts from the non-sclerotic subchondral bone also lead to the chondrocyte OA phenotype. Recent studies have shown that Wnt/β-catenin signaling plays an important role in OA occurrence and development (Wang et al., 2019). Activation of the pathways are considered to contribute to the destruction of the articular cartilage by enhancing catabolic events or contributing to the loss of the chondrocyte phenotype and being associated with extracellular matrix degradation (Usami et al., 2016; Maruotti et al., 2017). Therefore, it is likely that the Wnt activation of osteoblasts caused by hypoxia was an important mechanism for the OA-like shift of chondrocytes.

To verify the role of Wnt/β-catenin signaling in OA diagnosis, we detected β-catenin and SOST serum levels of patients with and without OA. Interestingly, OA patients had higher serum β-catenin levels and lower SOST levels, but no significant difference was found. However, the OA patients had significant lower serum levels of DKK-1, another typical antagonist of Wnt/β-catenin signaling that can be derived from synovial cells, articular cartilage chondrocytes, and subchondral bone osteoblasts in OA (Weng et al., 2009; Weng et al., 2010). The remarkable reduction of the antagonist of the Wnt pathway indicates that the pathway is activated in patients with OA. Consistent with our data, previous studies have shown that elevated circulating levels of DKK-1 are moderately associated with the severity of joint damage in knee OA (Min et al., 2017). Thus, our findings demonstrate that the Wnt/β-catenin signaling pathway may be potentially valuable for OA diagnosis; however, the precise mechanism underlying the interactions between these three factors in OA pathogenesis and development requires elucidation in larger longitudinal studies.

## Conclusion

In summary, the findings of the current study show that the subchondral bone of patients with OA exhibits signs of hypoxia. Furthermore, we showed that hypoxia can activate the Wnt pathway in primary osteoblasts isolated from the non-sclerotic subchondral bone of patients with knee OA. Above all, this study first demonstrated that under a hypoxic environment, osteoblasts from the subchondral bone are able to lead to a typical OA-like phenotype of articular chondrocytes, and this was achieved probably *via* activating the Wnt/β-catenin signaling pathway. These findings strengthen the hypothesis of a deleterious biochemical communication between subchondral bone and cartilage under subchondral bone hypoxia, which contributes toward our understanding of OA pathogenesis and aids the discovery of promising strategies for OA treatment.

## Data Availability

The original contributions presented in the study are included in the article/Supplementary Material; further inquiries can be directed to the corresponding authors.

## Abbreviations

ACAN, aggrecan; AS, late-stage subchondral bone; COL2AI, type II collagen; DKK-1, Dickkopf-related protein; DMOADs, disease-modifying osteoarthritis drugs; DMEM, Dulbecco’s modified Eagle’s medium; FBS, fetal bovine serum; HCM, conditioned medium from hypoxia; Hif, hypoxia-inducible factor; IOD, integrated optical density; IHC, immunohistochemistry; NCM, conditioned medium from normoxia; NS, early-stage subchondral bone; MMP13, matrix metalloproteinase 13; MMP3, matrix metalloproteinase 3; OA, osteoarthritis; PBS, phosphate-buffered saline; RT-PCR, reverse transcription-polymerase chain reaction; SOST, sclerostin; TKA, total knee arthroplasty.
